# Protocol for development of a reporting guideline (TRIPOD-Code) for code repositories associated with diagnostic and prognostic prediction model studies

**DOI:** 10.1186/s41512-025-00217-4

**Published:** 2026-02-10

**Authors:** Tom Pollard, Thomas Sounack, Catherine A. Gao, Leo Anthony Celi, Charlotta Lindvall, Hyeonhoon Lee, Hyung-Chul Lee, Karel G. M. Moons, Gary S. Collins

**Affiliations:** 1 MIT Institute for Medical Engineering and Science, Massachusetts Institute of Technology, Cambridge, MA USA; 2 Department of Biostatistics, Harvard T.H. Chan School of Public Health, Boston, MA USA; 3 Dana-Farber Cancer Institute, Boston, MA USA; 4 Northwestern Feinberg School of Medicine, Chicago, Illinois USA; 5 Harvard Medical School, Boston, MA USA; 6 Healthcare AI Research Institute, Seoul National University Hospital, Seoul, Korea; 7 Seoul National University College of Medicine, Seoul, Korea; 8 Julius Center for Health Sciences and Primary Care, University Medical Center Utrecht, Utrecht University, Utrecht, Netherlands; 9 Department of Applied Health Sciences, School of Health Sciences, College of Medicine and Health, University of Birmingham, Birmingham, UK; 10 NIHR Birmingham Biomedical Research Centre, University Hospitals Birmingham NHS Foundation Trust and University of Birmingham, Birmingham, UK

## Abstract

**Introduction:**

The Transparent Reporting of a multivariable prediction model of Individual Prognosis Or Diagnosis (TRIPOD) statement was published to improve the reporting and critical appraisal of prediction model studies for diagnosis and prognosis. This paper describes the processes and methods that will be used to develop an extension to the TRIPOD statement (TRIPOD-Code) for the management of code associated with prediction model studies. TRIPOD-Code focuses specifically on the transparent reporting of analytical code used in prediction model studies, including code for data preprocessing, model development, and model evaluation.

**Methods and analysis:**

TRIPOD-Code will be developed following published guidance from the EQUATOR Network and will comprise five stages. Stage 1 will involve a methodological review of how code availability is reported in published prediction model studies. In Stage 2, we will consult a diverse group of key stakeholders using a Delphi process to identify items to be considered for inclusion in TRIPOD-Code. Stage 3 will consist of virtual consensus meetings to consolidate and prioritise the key items. Stage 4 will involve developing the TRIPOD-Code checklist. In the final stage, Stage 5, we will disseminate TRIPOD-Code via journals, conferences, blogs, websites (including TRIPOD and the EQUATOR Network) and social media. TRIPOD-Code will provide researchers working on prediction model studies with a reporting checklist and accompanying guidance to promote code completeness and availability.

**Ethics and dissemination:**

This study has been determined to be exempt from ongoing IRB oversight by the Massachusetts Institute of Technology Committee on the Use of Humans as Experimental Subjects (COUHES) under Exempt ID: E-6675. Findings from this study will be disseminated through peer-reviewed publications.

## Background

Prediction models that estimate the health outcomes of an individual are widely available in the biomedical literature. These models are designed to support, for example, clinical decision-making or lifestyle management by generating predictions about a current condition (diagnostic models) or a future event (prognostic models) [1, 2]. To assess and apply these models in daily practice, clear and complete reporting is essential. This need led to the development of the Transparent Reporting of a multivariable prediction model for Individual Prognosis Or Diagnosis (TRIPOD) Statement, which provides guidance for the reporting of prediction model studies [3, 4].

While early prediction models were typically based on regression methods, recent years have seen a rapid increase in the use of machine learning and other forms of artificial intelligence. In response, the TRIPOD+AI guidelines (www.tripod-statement.org) were published in 2024 to offer unified reporting recommendations for studies using either traditional statistical models or machine learning approaches [5]. A key addition in TRIPOD+AI was a new “Open Science” section, which includes an item encouraging researchers to report whether the analytical code is available [6].

However, sharing code in a simple and standardised way to facilitate reproducibility remains challenging. In the context of prediction model studies, different types of code serve distinct purposes, including analytical code used for data cleaning and model development, evaluation code used to assess model performance, and implementation code required for applying a trained model to new data. TRIPOD-Code primarily targets the transparent reporting of analytical and evaluation code that underpins published results, while recognising that model implementation code may be shared separately as part of model specification.

There is little available guidance on what makes code sufficiently clear and structured for sharing, either for prediction model studies or for research code in general. As a result, even when code is made available, the usability and completeness of what should be shared can vary considerably [7]. Important details are often missing, including how the code is structured, what dependencies it requires, any license restrictions, any testing details, or how it will be archived for long-term access. Without this information, it becomes difficult for others to reproduce results, assess the quality of a study, or use or extend the code for future research.

The TRIPOD-Code extension is intended to address this gap by providing a checklist of minimum reporting requirements for code quality and availability in prediction model studies. The checklist will cover key elements such as documentation of software dependencies, specification of license terms, and whether the code is modular, tested, or reproducible. While its primary aim is to improve transparency through reporting, our hope is that TRIPOD-Code will indirectly encourage good coding practices by serving as a point of reference for the community. We note that the software engineering field has developed extensive guidance on good coding practice, documentation, and testing. TRIPOD-Code is not intended to replace this literature, but to draw on and contextualise the most relevant principles for prediction model studies.

By standardising expectations around code availability and quality, TRIPOD-Code aims to support reproducibility, facilitate independent validation and implementation, and ultimately increase the impact of prediction models in healthcare. Some checklist items may be reported in the main text of the research article, while others may be addressed within the associated code repository or supplementary materials.

## Focus of TRIPOD-Code

TRIPOD-Code is intended to complement the existing TRIPOD guidance by addressing reporting considerations specific to analytical code and computational reproducibility, rather than revising or merging into the core TRIPOD checklist. In line with earlier TRIPOD guidelines, the focus of TRIPOD-Code is on reports of projects in which a multivariable prediction model is developed, updated or validated using any statistical or machine learning technique, with a particular emphasis on the transparent reporting of code availability and computational reproducibility. Conforming to the original TRIPOD definition, a multivariable prediction model is any combination or equation of two or more predictors that is used for individualised predictions to estimate an individual’s probability of having (diagnosis) or developing (prognosis) a particular health outcome or state.

Predictors may take any form and can be derived from patient history, physical examination, diagnostic, prognostic or monitoring tests, as well as treatments received. Outcomes may also take any form (dichotomous, categorical, continuous) and include, for example, the presence or absence of a condition (diagnostic outcome), short-term outcomes (e.g., hospital mortality or postoperative complications), or long-term prognostic outcomes (e.g., 1-year occurrence of treatment complications, 5-year occurrence of metastases, or overall survival). TRIPOD-Code will address prediction model studies from all medical care settings (public health, primary, secondary, tertiary, and nursing home care) and among all corresponding target populations (healthy individuals, suspected individuals, and those with a confirmed disease).

While analytical code is central to computational reproducibility, full reproducibility typically also depends on access to the underlying data. TRIPOD-Code focuses specifically on reporting the availability and structure of code and aims to enhance transparency even in settings where data sharing is not feasible. When data cannot be shared (for example due to privacy, governance, or licensing considerations), we note that providing demo or synthetic data can offer substantial benefits by enabling others to understand, test and reuse the code.

Although TRIPOD-Code is designed with clinical prediction models in mind, we anticipate that many or most of the recommendations will be applicable to any field where scientific code underpins research findings. The principles of transparency, reproducibility, and accessibility are broadly applicable and may benefit a wide range of scientific disciplines that rely on statistical and computational methods.

A key focus of TRIPOD-Code is to improve transparency in the *reproducibility* of studies, particularly through the availability of analytical code. This includes code used for data cleaning, feature engineering, model building, and evaluation. Making this code accessible and well-documented is essential for verifying results and facilitating reuse by other researchers. This focus aligns with Item 18f of the TRIPOD+AI checklist:

“Item 18f (Code sharing): Provide details of the availability of the analytical code. This relates to the analysis code, for example, any data cleaning, feature engineering, model building, evaluation.”

While reproducibility is the primary focus of TRIPOD-Code, we acknowledge the importance of enabling others to reuse or apply a developed model in new contexts. Item 22 of the TRIPOD+AI checklist encourages reporting of details such as model files and APIs for this purpose:

“Item 22 (Model specification): Provide details of the full prediction model (e.g., formula, code, object, application programming interface) to allow predictions in new individuals and to enable third-party evaluation and implementation, including any restrictions to access or re-use (e.g., freely available, proprietary). This relates to the code to implement the model to get estimates of risk for a new individual.”

TRIPOD-Code will offer guidance in this area; however, we recognize that the growing use of complex AI models has introduced new challenges, including the need to sometimes manage model artifacts (e.g., trained weights or checkpoint files) separately from the codebase. As such, TRIPOD-Code will treat model sharing as distinct from code sharing, and may refer to complementary practices or emerging standards specific to model specification and availability.

## Methods/design

TRIPOD-code will be developed following published guidance from the EQUATOR Network [8]. We will develop the guideline in five stages, designed to ensure the guideline reflects both empirical findings and expert consensus:

- Meta review to establish the quality of current reporting regarding code availability
- Delphi exercise
- Consensus meeting
- Development of the guidance statement
- Guideline dissemination

We have registered our intent to develop the TRIPOD extension for code availability on the EQUATOR Network website (www.equator-network.org) and the TRIPOD website (www.tripod-statement.org).

### TRIPOD-Code working group

The TRIPOD-Code working group will include: (1) an executive committee, (2) an advisory and working group, and (3) a large international Delphi panel.

The TRIPOD-Code executive committee (TP, TS, LAC, CL, HL, HCL, KGMM and GSC) will be responsible for the leadership and coordination of all processes involved in the development and dissemination of the TRIPOD-Code guideline. The executive committee comprises lead authors of the original TRIPOD reporting guidelines, along with prediction model experts and researchers with a focus on code availability and reproducibility in machine learning. Key stakeholders for Stage 2 (the Delphi survey) will be identified and approached to participate, and a subset of these stakeholders will take part in Stage 3 (the consensus meeting).

The term key stakeholder refers to a cross-sector participant (from both industry and the public sector) who falls into at least one of the following categories:

1. Researchers with experience in prediction models in healthcare settings using statistical or machine learning methods.
2. Researchers with expertise in code transparency and reproducibility.
3. Assessors and approvers of statistical and machine learning models for healthcare applications, such as regulatory assessors and members of ethics committees.
4. Journal editors and reviewers.
5. Commissioners of research grants, such as funding agencies.
6. Consumers of research results, including healthcare providers and patients.

### Stage 1: meta review of code associated with prediction model studies

A meta review is underway to evaluate code associated with published studies that develop, validate, or update prediction models in the medical domain using the TRIPOD or TRIPOD+AI guidelines, with a particular emphasis on code availability and computational reproducibility. The review will assess adherence to the TRIPOD Statement on code sharing, and identify specific issues related to code transparency and reproducibility that are not currently addressed by TRIPOD.

Undertaking this meta review serves two main purposes: (1) to understand the extent of code availability and information reported to describe code availability in clinical prediction studies, particularly those citing the TRIPOD and TRIPOD+AI guidelines, and (2) to identify candidate reporting items for consideration in the TRIPOD extension focused on code availability. The executive committee will identify preliminary items from the literature to be considered in Stage 2 (the Delphi study) for inclusion in the eventual TRIPOD-Code checklist.

### Stage 2: Delphi exercise

We will perform a Delphi survey among a large and diverse international network of relevant stakeholders, with a maximum of three rounds, to help decide on items that should be included in the new TRIPOD-Code checklist.

#### Design

The Delphi process will comprise a series of rounds in which panelists independently and anonymously evaluate and achieve consensus on the inclusion or exclusion of the proposed reporting items, with particular attention to those related to code availability and reproducibility. This process will be repeated for a maximum of three rounds. After each round, participants will receive structured feedback from the previous round to help reconcile individual opinions and foster group consensus. Items achieving a high level of agreement ($\ge 70\%$) will be taken forward to the consensus meeting in Stage 3.

#### Selection of potential items

The list of candidate items for TRIPOD-Code will be collated by the executive committee, drawing on:

- The results of the meta review;
- Other available studies on scientific code and prediction;
- Expert recommendations from the Delphi panel.

Relevant methodological guidance and papers will also be reviewed to identify additional candidate reporting items. Pre-selection will involve categorizing items into those to be further considered, those to be provided as optional guidance, or those not to be considered for inclusion. Delphi participants will have the opportunity to view and provide feedback on each item in every round and to suggest new items.

#### Recruitment process and participants

Delphi participants will be identified through the professional networks of the executive committee, previous participation in TRIPOD and PROBAST (www.probast.org) related Delphi exercises, responses to relevant publications where TRIPOD-Code was announced, and via social media outreach.

We will invite international participants with diverse roles (e.g., researchers, healthcare professionals, journal editors, funders, policymakers, healthcare regulators, and end users of prediction models) from a variety of settings (e.g., universities, hospitals, primary care, biomedical journals, non-profit organizations, and for-profit organizations). Invitations will be sent via personalized email describing the development of the TRIPOD-Code extension, outlining its objectives, process, and timelines.

We plan to invite at least 200 participants to the Delphi survey with broad geographic diversity. For each round, the survey will remain open for 2 weeks, with a reminder email sent 1 week after the initial invitation. In the second round, additional participants may be recruited to ensure fair representation of all key stakeholders. Participants will provide their name, contact information, and a small set of demographic and professional characteristics to enable coordination of Delphi rounds and reporting of panel diversity. Free-text comments may be shared anonymously in the feedback summaries between rounds and in publications arising from the study. Any identifying information will be removed prior to sharing. All data will be stored securely on institutional servers at the Massachusetts Institute of Technology and only aggregate or anonymised results will be shared outside the study team.

Informed consent will be obtained using an online consent form, and participants can withdraw at any time. Individuals who opt out will be removed from subsequent rounds. Participants will remain anonymous to each other and will not see individual responses.

#### Procedure for selection of items

Participants will be asked to consider the following guiding principles when reviewing existing, new, or modified items for inclusion:

1. Reproducibility: The reporting of the item should enable others to replicate the study’s findings based on the provided code and methodological details.
2. Transparency and Quality: The item should facilitate the assessment of the study’s quality and transparency, enhancing its use in subsequent studies, reviews, and daily practice.
3. Broad Relevance: The item is likely relevant to nearly all prediction model studies.
4. Minimum Reporting Standards: The set of items should represent the minimum that should be reported in all studies developing (including updating) or validating a healthcare prediction model, with particular emphasis on code availability.

#### Round 1

Participants will rate each checklist item on a 5-point Likert scale regarding their agreement with its inclusion in the TRIPOD-Code extension (1 = strongly disagree, 2 = somewhat disagree, 3 = I don’t know, 4 = somewhat agree, 5 = strongly agree). A free-text box will be provided for each item to allow participants to justify their decisions or suggest wording changes, and an additional free-text box will be available at the end of the survey for general comments or to propose new checklist items. The survey will be pilot-tested for usability and clarity with a small group of individuals familiar with scientific code (but not involved in the TRIPOD-Code development) and revised accordingly based on their feedback.

#### Round 2

The same participants from Round 1 will be invited to Round 2. They will receive their individual Round 1 responses along with an anonymized summary of group ratings and comments for each item. Using the same format as in Round 1, participants will review each item – including any new items suggested in Round 1 – and indicate their agreement with its inclusion in the TRIPOD-Code checklist, taking into account the structured feedback provided. Participants who did not respond in Round 1 will also be invited to Round 2 and provided with the anonymized group summary. Items that reached a high level of agreement (scoring 4 or 5 by $\ge 70\%$ of respondents) in Round 1 will be presented for information purposes only, with no further voting, though participants will still be invited to comment. A third Delphi round will be conducted if the executive committee deems necessary.

#### Results from the Delphi survey

Item ratings will be summarized for the entire panel (e.g., frequency and proportion of ratings) along with a narrative summary of the findings, comments, and suggestions. The executive committee will review the results from all rounds. Items for which consensus was not achieved after the second round will be further discussed by the executive committee and considered for discussion at the subsequent consensus meeting in Stage 3.

### Stage 3: consensus meeting

A virtual consensus meeting will be held with the objective of discussing the results from the Delphi exercise and finalizing the items to be included in the TRIPOD-Code reporting guideline. The meeting will include members of the executive committee and a purposively selected subset of Delphi participants. Selection will seek to ensure representation across key stakeholder groups and will prioritise individuals with demonstrated engagement in the Delphi process and relevant methodological expertise. We expect approximately 15 participants to contribute to the virtual consensus meeting, in addition to executive committee members. Additional experts who did not take part in the Delphi may also be invited, at the discretion of the executive committee.

#### Procedure

The executive committee will prepare the agenda and relevant materials (e.g., results from the meta review and the Delphi exercise) and share these in advance with all attendees. During the meeting, executive committee members will facilitate a structured discussion on the rationale behind each candidate reporting item identified during the Delphi exercise. Participants will have the opportunity to discuss each proposed reporting item and then vote on its inclusion. An item will be retained for inclusion in the final TRIPOD-Code checklist if it achieves at least 70% support from the consensus meeting participants. Items falling just below this threshold (e.g., 60–69%) will be flagged for further consideration. During the consensus meeting, the group will agree on a draft list of reporting items for the final TRIPOD-Code extension. While specific wording will not be finalized during the meeting, the general intent and meaning of each item will be discussed. Plans for dissemination will also be addressed at the conclusion of the meeting.

Following the meeting, the executive committee will deliberate on any borderline items and propose final decisions. Because some candidate items may be partially redundant or conceptually overlapping, endorsement rates will not be interpreted in isolation. The executive committee will review such items collectively, taking into account whether two or more overlapping items together reflect strong support. In these cases, items maybe merged, reworded, or combined prior to finalisation of the checklist. These decisions will then be communicated back to the consensus meeting participants for approval or further discussion, ensuring transparency and group endorsement of any modifications.

#### Pilot testing

We will invite authors of prediction model studies in the medical domain, doctoral students participating in prediction model courses and workshops, and peer reviewers and editors of journals that frequently publish such studies to pilot a draft version of the TRIPOD-Code checklist. Feedback will be sought regarding the clarity and interpretability of the item wording, with particular attention to whether any items are ambiguous or difficult to understand.

### Stage 4: development of the TRIPOD-Code statement

The executive committee will lead the development of the TRIPOD-Code reporting guideline, based on the list of items agreed during the consensus meeting (Stage 3). The executive committee will reserve the right to revise the checklist items during the development process, for example as a result of feedback from pilot testing. After revisions, a full draft manuscript, including the finalized checklist and a description of its development process, will be circulated to all consensus meeting participants for their comments, further refinement, and approval.

### Stage 5: guideline dissemination

The dissemination strategy will be informed by discussions at the consensus meeting. We will aim for simultaneous publication in key journals to reach diverse readerships. To increase visibility and promote uptake, the TRIPOD-Code checklist will be published open access and made available on the TRIPOD website alongside other TRIPOD extensions (www.tripod-statement.org), and indexed on the EQUATOR Network website (www.equator-network.org). Social media channels will also be utilized to disseminate the extension widely. The executive committee, and consensus meeting participants will be encouraged to publicize the TRIPOD-Code statement at key conferences and courses.

#### Publication plan

We expect the following publications to arise from the TRIPOD-Code initiative:

1. Publication 1: Study protocol.
2. Publication 2: Review paper.
3. Publication 3: TRIPOD-code statement.

All participants of the online consensus will be offered authorship on the TRIPOD-code statement, providing they meet the requirements for authorship.

## Conclusion

TRIPOD-Code will guide researchers in reporting the completeness and availability of code in prediction model studies in any healthcare setting. Additionally, we anticipate that the checklist will provide a framework that promotes better practice in code sharing, and which assists reviewers, editors, policymakers, and end users in understanding the methodologies and findings, ultimately improving research quality and efficiency.

## Data Availability

No datasets were generated or analysed during the current study.
